# Effect of product-based pedagogy on students’ project management skills, learning achievement, creativity, and innovative thinking in a high-school artificial intelligence course

**DOI:** 10.3389/fpsyg.2022.849842

**Published:** 2022-08-26

**Authors:** Zehui Zhan, Wenyao Shen, Wenkai Lin

**Affiliations:** ^1^School of Information Technology in Education, South China Normal University, Guangzhou, China; ^2^Key Laboratory of Brain, Cognition and Education Sciences, Ministry of Education, Guangzhou, China; ^3^Teacher Education College of Guangdong-Hong Kong-Macao Greater Bay Area, South China Normal University, Guangzhou, China; ^4^Department of Curriculum and Instruction, The Education University of Hong Kong, Hong Kong, Hong Kong SAR, China

**Keywords:** product-based pedagogy, creativity, innovative thinking, 7P model, high school, introductory artificial intelligence course

## Abstract

The purpose of this study is to explore the effectiveness of product-based pedagogy (PBP) on students’ creativity and innovative thinking in artificial intelligence (AI) education. A seven-step model (i.e., phenomenon, problem, plan, prototype, product, presentation, price) in accordance with PBP was proposed, in which the key function of the product as a linkage between creativity and innovation was emphasized. A total of 209 students from a major high school in South China were randomly assigned to a treatment group with PBP and a control group with direct instruction. Results indicated no significant difference was found in students’ learning performance; however, students in the treatment group performed significantly better than the control group in terms of students’ project management skills, creativity, and innovative thinking. This research validates the feasibility and effectiveness of the PBP and highlights its advantages for high-school AI education, which indicates a new direction for cultivating creative and innovative talents.

## Introduction

In recent years, the rapid development and the disruptive potential of artificial intelligence (AI) are a global trend and call on innovative talents in the future society. Various countries have issued corresponding education policies to actively welcome the opportunities and challenges brought by AI technologies and prepare for the arrival of the new era (Zhou et al., 2021). Despite this, there is a limited discussion of pedagogical approaches utilized in high school that are applicable to teaching AI courses.

There are inconsistent findings on the effects of different pedagogies in AI courses. According to the PISA report (OECD, 2016), teacher-directed instruction is significantly associated with improved science performance in schools. We were surprised to find that inquiry-based instruction was reported to be negatively related to students’ learning performance in science and technology. However, opposite results were obtained in some empirical studies, which claimed that problem-based and inquiry-based learning is more conducive to helping students achieve better performance in these courses (Liu et al., 2018; Zhan et al., 2022).

Moreover, AI education at the high-school level emphasized the importance of cultivating creativity and innovation among students in order to keep up with future technological advancements. Notably, there are some differences between these two abilities: creativity involves curiosity, risk-taking, challenge, and imagination (Williams, 1980; Sternberg, 2006; Runco, 2014), emphasizing the generation of creative ideas and developing original conception of new things (Westwood and Sekine, 1988); innovation involves decision-making, feasibility, practicality, effectiveness, representing the market demand, and the value of the product (Fagerberg et al., 2005; Clydesdale, 2006; Schumpeter et al., 2017).

Both creativity and innovative thinking involve convergent and divergent thinking, and they are closely related to the process of generating ideas and interacting frequently. Given the aforementioned commonalities and differences in connotations, creativity and innovative thinking have an important connection: the creation of products through thinking. The product plays an essential role in the pedagogy, which triggered students to carry out meaningful creations according to social needs, starting from the problem to be solved and ending up in the form of products (Yulastri et al., 2017; Guo et al., 2020).

In AI courses, the product enables students to encounter real engineering problems and motivates them to develop original ideas and transform ideas to market value. Therefore, we try to propose a special kind of project-based learning, namely product-based pedagogy (PBP) in this study, regarding products as the connection between creativity and innovative thinking (Zhan et al., 2019), with market demand driving the process of product design, implementation, and promotion iteratively. In PBP, products are the basis for the formation of market value by innovative thinking, which is also the result of creativity. The purpose of this paper is to propose a feasible PBP pedagogy and examine its effect on students’ project management skills, learning achievement, creativity, and innovative thinking in a high-school AI course, in order to provide a model case and empirical evidence for adopting PBP in AI education.

## Literature review

### Creativity and innovative thinking

The concept of creativity encompasses core ideas such as “creating something new,” “expressing something in a novel way,” “finding new connections,” or “evoking pleasant surprises” (Maley, 2003; Lin et al., 2022). Creativity can be seen as the ability to create and the personality of being creative, which Krathwohl (2002) describes as “putting elements together to form a novel, coherent whole or to produce an original product.” According to Torrance (1974), creativity is the ability to think in innovative ways to solve problems and produce original, valuable ideas, which is a complex composition affected by both psychological and environmental factors. Sternberg (2006) argued that creativity is the result of the interaction of five psychological resources (i.e., intelligence, knowledge, thinking style, personality, and motivation). Later on, these factors were further proposed as three major elements (i.e., work motivation, domain skills, and related creative skills) (Amabile, 2011; Amabile and Pratt, 2016). In sociocultural definitions, creativity is the collaborative action of creating a product judged to be innovative, appropriate, useful, or valuable, whereas, in the individualistic definitions, it is the manifestation of a new psychological combination (Sawyer, 2011). Based on different theoretical frameworks, Runco and Jaeger (2012) proposed a standard definition (SD) that creativity requires both originality and effectiveness, which was regarded as a relatively static perspective. Later on, Corazza and Lubart (2020) proposed a definition claiming “Creativity is a context-embedded phenomenon requiring potential originality and effectiveness,” which emphasized the dynamic character of the creative process (Corazza and Lubart, 2021).

Scholars have also elaborated on the concept of creativity from the sociocultural and developmental perspectives. For example, Vygotsky (2004) believed that creativity is inherent to human beings. Gruber (2020) proposed an evolving systems perspective on creative lives that was developmental, interactive, and context-sensitive. With Clapp and Hanchett Hanson (2019)’s participatory framework, young people can effectively engage in creative activities that consider their interests and experiences. Glăveanu (2014) proposed a distributed creativity theory that stresses the dynamic, sociocultural, and developmental nature of creativity. Csikszentmihalyi (2015) believed that creativity is never solely the result of individual activity, it is the combined effect of three main forces (i.e., social institutions, stable cultures, and individuals).

It is increasingly apparent that creativity is a necessary skill for the twenty-first century and that it can be incorporated into the curriculum from an early age (Vygotsky, 2004; Said-Metwaly et al., 2017; Beghetto, 2019). Generally, creativity is recognized as a cognitive and emotional endeavor (Fuchs et al., 2007; Ivcevic et al., 2007), and the discovery learning process enhances creative performance to help learners manipulate surroundings and generate new ideas (Munro, 2011; Carlsen and Välikangas, 2016). In cognitive neuroscience, creative thinking is thought to be a process in which cortical regions form and restore connections constantly (Zhang and Bai, 2006). Besides, the study of metacognition has been widely discussed and scholars believed that it is important to help students metacognitively understand the concept of creativity, thus increasing creative awareness and the quality of creative products (Davis, 1991; Hargrove and Nietfeld, 2015).

There are many ways to conceptualize creativity (e.g., approaches that focus on cognitive neuroscience, environmental factors, sociocultural processes, metacognition, learning theory, developmental psychology, etc.). In the field of AI, some scholars combined creativity with machine learning to emphasize the role of inventing new ideas to support the process of machine creation (Wendrich, 2020). It was found that the AI learning process mainly focused on clear reasoning but ignored the creative emergence of new ideas, especially the integration of AI technology in creativity (Wegerif et al., 2009). According to Park et al. (2021), creative problem-solving is one of the most essential competencies in engineering education. By applying entrepreneurship and innovation education to the construction of AI courses, creativity can provide a new understanding of the relationship between scenarios, goals, and solutions to problems (Tan, 2020).

Innovation is a terminology that dates back to the Greeks and the Romans in Western culture and is probably even older in Eastern culture (Saxena, 1993; Zambon, 2008; Godin, 2015). However, the formal appearance of “innovation” as an academic concept originated in Schumpeter’s “Theory of Economic Development,” which defined it as a change in the production function and recombination of existing resources (Schumpeter, 1912). Similarly, Westwood and Sekine (1988) regarded innovation as the process of converting an invention into a useful product or system. Roberts (1988) described it as invention and diffusion, in which invention refers to the attempts of creating new ideas, whereas diffusion refers to the development of these ideas, such as their application, transfer, and evaluation of success. Romer (1992), a representative of the new economic growth theory, asserted that innovation is essentially a mechanism that enables the generation and application of new designs or ideas.

Although the term “innovation” has been extended from the field of economics to various industries, its connotation remains to emphasize the process of generating new value, which can be either a new product or a new combination of factors. When a creation, a work, or an invention has market value, it becomes an innovation (Wang and Zheng, 2017). Therefore, innovation describes a process that is novel and provides a measurable economic benefit (Xie and Zhuang, 2006). In this vein, Jones (2015) regarded it as the first application of an invention or the first commercialization of scientific research outputs. Apart from this, innovation also emphasizes putting innovative ideas into action (Fagerberg et al., 2005). Innovative thinking is a cognitive process that leads to innovation (Lindfors and Hilmola, 2016; Keller-Bell and Short, 2019). It stimulates the realization and accomplishment of new ideas (Anderson et al., 2014; Barak and Usher, 2019).

A supportive environment and conducive conditions are essential to cultivating innovative thinking. According to the theory of innovation ecology, the link between the subjects and their environment is vital. By integrating the creative atmosphere in schools and the innovative environment of the enterprises and society, an effective mechanism of industry–university alliances can be developed, which is crucial to cultivating innovative talents (Lin, 2018; Mei et al., 2022).

Therefore, creativity pursues “novelty,” “original creation,” and the “unprecedented,” whereas innovation emphasizes commercial elements, with “feasibility,” “practicability,” “effectiveness,” and “decision-making.” From the aforementioned similarities and differences in connotation, it can be concluded that creativity and innovative thinking share a significant link, which is the creation of a product. As a carrier of the transformation from creativity to innovative thinking, the product is the result of creativity, but it also serves as a basis for innovative thinking to form market value. Therefore, product creation is a critical step in the creativity–innovative thinking linkage. Various factors need to be considered during the complex, diverse, and iterative process of the final product creation (Zhan et al., 2019; Lin et al., 2022). To summarize, although the concept of creativity varies widely, for this study, creativity will be defined as an intellectual quality that generates original, novel, and socially meaningful products, and innovative thinking will be defined as a cognitive level that enables the formulation, invention, and construction of products with sufficient market value.

### Product-based pedagogy

Project-based pedagogy originated in the architectural and engineering education movement that emerged in Italy in the late sixteenth century (Knoll, 1997). Kilpatrick (1918) first mentioned PBL based on Dewey’s theory of experience. It is generally generated by a problem and leads to a project plan to deal with the challenge (Blumenfeld et al., 1991). Compared to traditional instruction, PBL includes more autonomy, choice, and unsupervised working opportunity for students (Yousuf et al., 2010). Due to its features such as challenging students with real-world problems and giving students responsibility for learning, PBL has attracted tremendous attention around the world (Barrows, 1994; Frank and Barzilai, 2004).

PBP is an extension of PBL (Ragan et al., 2009), which emphasizes a tangible product as one of the project outputs, and potentially generates students’ creativity and innovative thinking (Zhan et al., 2019). As Prince and Felder (2006) have pointed out, a final product is crucial to the achievement of learning goals. By focusing on product design and development to form solutions to the problems, PBP enables students to become active learners to achieve learning goals and promotes social interaction and meaningful learning.

Pedagogical models such as design thinking and maker education have gained increasing attention in recent years as inquiry-based approaches that bring insights to PBP. According to Carroll et al. (2010), design thinking is a way to develop students’ creative confidence by encouraging them to participate in hands-on projects that enhance empathy, establish ideas, and promote positive problem-solving. Design thinking in education is also reflected in students’ ability to recognize others’ needs and respond when interacting with other students in the design process (Wells, 2013). Maker education aims to design, build, modify, and repurpose objects to produce a “product” that can be consumed, interacted with, or demonstrated by using traditional craft techniques or digital technologies (Veldhuis et al., 2021). In essence, maker education allows students to practice hands-on activities and encourages their creative realization and expression in the maker space. Comparatively, design thinking highlighted the application of empathy and the follow-up steps of defining, ideating, prototype, and testing (Wu et al., 2022); maker education focuses on the idea materialization and the iteration process (Goldman and Kabayadondo, 2016). Both pedagogies pay less attention to the market value of the product and neglect further integration of business expertise and marketing skills learning, which is crucial for innovative thinking training.

In Table 1, we compare PBP with the other pedagogies (i.e., direct instruction, PBL, design thinking, and maker education) from the perspectives of concept, feature, and steps, so as to sum up the features of PBP. As can be seen, PBP is different from the other pedagogies listed in the table. For example, “direct instruction” is a teacher-centered strategy in which the teacher is the primary source of information. Learning occurs when students interact directly with the ideas, skills, and information presented by the teacher, and teaching is effective when it involves the direct communication of facts, rules, and sequences of actions to students, which is different from PBP which encourages students to create products that meet real-world needs. Compared to PBL which generally adopts a question or problem that serves to organize activities (Blumenfeld et al., 1991), PBP is inherently product-oriented and emphasizes the product as a creative and innovative learning outcome. Moreover, PBP integrates the advantages of design thinking and maker education. It integrates empathy into the problem discovery process from situated phenomena, allows students to think from others’ perspectives, and facilitates cooperation around a shared vision of product design and utility analysis. Furthermore, PBP emphasizes the need to balance the current performance of products with their potential for the future, while considering the effects of product iteration and generation. The tangible product allows students to demonstrate the market phenomenon, discover the values, and transform creativity into innovative thinking.

**TABLE 1:** Comparison of PBP and other pedagogies.

	Direct instruction	PBL	Design thinking	Makers education	PBP
Connotation	A teacher-centered pedagogy in which the teacher gives lectures or demonstrates exercises and presents the information in clear steps, then students follow the instructions to reinforce their knowledge and skills.	A general learner-centered pedagogy that organizes learning around projects and focuses on facilitating inquiry, problem-solving, and investigation around challenging problems	A pedagogy for meaningful and effective design through hands-on activities helps to build empathy, foster action, encourage ideas, and promote positive problem-solving	A pedagogy for generating a creative idea and materializing it by making, tinkering, and emphasizing the construction, modification, and/or reuse of material objects applicable to craft techniques or digital technologies.	A pedagogy that focuses on product design and development to solve real-world problems and discover the market value, using products as a vehicle to promote creativity and innovative thinking
Features	1. An explicit step-by-step strategy. 2. Development of mastery at each step in the process. 3. Strategy (or process) corrections for student errors. 4. Use adequate examples. 5. Teachers provide feedback and guidance.	1. Driving Questions 2. Situated Inquiry 3.Collaborations 4. Using Technology Tools To Support Learning 5. Creation Of Artifacts	1. User-centered, empathy-driven approach designed to create solutions 2. Based on a human-centered experience 3. Guided prototyping through a “test-and-learn” cycle	1. Advanced technical equipment support system 2. Open source, free, and sharing maker culture 3. Quasi-real situation learning mode	1. Emphasizing tangible products as the project outcome and as a carrier to promote students’ creativity and innovative thinking 2. Motivate students with phenomena for problem identification and solving 3. Emphasize iteration and value assessment
Steps	Breaking instructional tasks into small steps through explicit teacher-led instruction	Four processes: planning, designing, producing, and revising	Five stages of action: empathy, definition, conceptualization, prototyping, and testing	Four fundamental phases: preparation, experimentation, prototyping, and integration feedback	7P: phenomenon, problem, plan, prototype, product, presentation, price

### Effect of product-based pedagogy on learning outcomes

It is believed that PBP offers a certain advantage for students’ learning as it advocates student-centered activities and encourages learner-centered activities (Romero-Saritama, 2019). For example, it enables students to discover real engineering product problems (David and Larry, 2001) and seek solutions from the observed issues (Romero-Saritama, 2019), which is a learning outcome that meets social needs instead of simply memorizing basic knowledge (Hidayat, 2017). Besides, PBP was regarded as an active learning strategy (Rosales-Torres et al., 2020) and was reported to be effective in promoting student’s creativity (Kaufman et al., 2017; Widyastuti and Utami, 2018; Zhou et al., 2021), innovative skill development (Cannon and Leifer, 2001), and learning performance (Jeprimansyah et al., 2018). Mardin et al. (2018) argued that PBP was conductive to increase students’ learning achievement, involving the internalization of knowledge, skills, affections, and competencies through structured processes in science and technology education. Moreover, the previous study has reported that PBP offered a better opportunity for students to practice their project management skills (including time management skills, communication, and collaboration skills, etc.) during the problem-solving process (Shekar, 2007) and the creation of specific products, which can also be used as lesson examples to trigger students’ creativity and innovative thinking (Zhan et al., 2019).

However, some previous research also pointed out that PBP is not always superior to direct instruction. For example, some teachers took products as the standard of learning outcomes, worrying that students’ insufficient time management and self-management ability will affect the final shape of products, so they designed each project process in advance and gave students less choice (Wang, 2019). The student’s product also has some problems such as one-time molding without sufficient revisions and iterations, and little connection with disciplines curriculum standards (Condliffe, 2017). Another situation might be that PBP is not always applicable, because not all the projects could end up in the form of a product. For example, if the problem to be solved is a political or an ethical issue, it might not be suitable to generate a tangible product (Jia and Lin, 2014).

Given the debate that exists in literature, this study tried to establish a feasible model and adopt PBP in an AI course and examined its effect on students’ project management skills, learning performance, creativity, and innovative thinking, which are variables mentioned in previous research that yielded inconsistent conclusions. Therefore, this study might provide a practical case and empirical evidence on the method of applying PBP in AI education and effectively develop a new approach for cultivating innovative talents.

## Conceptual framework

### A 7P model for product-based pedagogy

The goal of PBP is to create a final product that identifies the challenge that must be solved at the outset, allows students to actively participate in the process, and delivers social value. In this approach, students are motivated to learn by creating products that meet real-world needs while also integrating diverse and innovative ideas into the product creation process (Ragan et al., 2009). As a vehicle for linking creativity and innovative thinking, product creation helps students to think creatively, and understand the conditions necessary for innovative ideas to be realized and transformed into market value. While students engage in the process, they can reconstruct their knowledge, enhance their ability to work with others, and improve confidence and interest in the project, with the final product being a concentrated expression of the various competencies they may develop.

The product orientation in PBP is reflected in the clear outcome requirements, as well as the construction of the product creation process (7P), which outlines the steps involved in fulfilling the task and product presentation. By integrating fragmented knowledge into a systematic one, students can identify problems from phenomena and ultimately create innovative products that can solve problems through prototyping.

The products in PBP can be determined based on a problem-solving approach, including physical products, research reports, design solutions, etc. Conceptualization of a product does not occur all at once, and prototype construction is planned and completed through iterations. The process from prototype to product is then consciously tested, and opportunities are identified from idea generation to product creation. Ultimately, by analyzing the direction of the subsequent sustainable development of the product through the presentation, the interactive and linkage cultivation of creativity and innovative thinking is realized.

**FIGURE 1:**
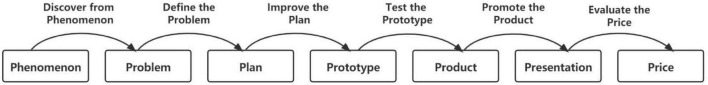
The 7P model of PBP.

As PBP emphasizes the organization of learning activities around product design, this study proposed a 7P model, consisting of seven steps starting with “P” (i.e., phenomenon, problem, plan, prototype, product, presentation, price). Using the 7P model, teachers should create the project situations based on real-life phenomena, so that students can pay attention to the actual situation rather than theoretical concepts, and then discover the core problems that need to be solved. Then, the students are guided to decompose the problems and explore potential directions for the solution, activating their ideas and creativity to develop a plan for the target product. According to the plan, the students collaboratively improve their ideas through practice and try to transform the idea into a conceptual prototype. After multiple iterations, the prototype is adjusted and finally leads to an entire product with a clear market value. Then after analyzing the unique feature and value of the product, students may illustrate the product in a simulated market environment through the presentation. Finally, based on the feedback from the presentation, students try to price the product in conjunction with the estimation of its value, which allows them to grasp the rules of the market and the business model. Figure 1 shows the 7P model of PBP.

### Applying the 7P model in artificial intelligence education

**FIGURE 2:**
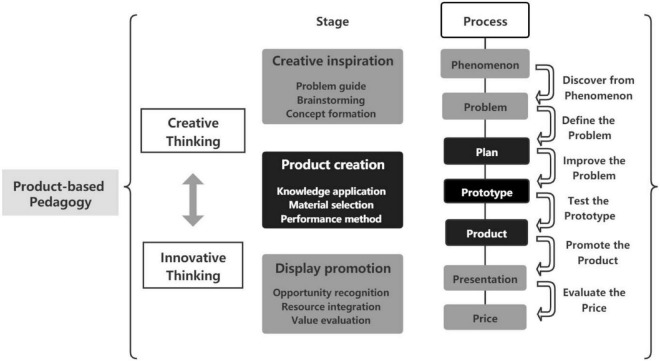
PBP in high-school AI education.

The goals of AI education are to assist students in understanding the features of AI and the method of applying the technologies in daily life. In a certain sense, AI technology represents the frontier of information technology. Students can obtain knowledge by using AI technologies to create a product (Liu et al., 2018). With the development of society, the trend of integrating education, science, and technology and the economy is becoming stronger, driving the demand for creative and innovative talents. AI courses contribute to product innovation by integrating key technologies that facilitate the achievement of a complete innovation chain from idea to creation, then to application. In accordance with the characteristics of AI education, as well as the cognitive characteristics of high-school students, this study proposes a 7P model in AI education, in which the PBP is divided into three stages as shown in Figure 2.

The first stage is creative inspiration (phenomenon setting and problem discovery). Students are guided in discovering problems from phenomena in a specific problem situation, and relevant examples are presented, which not only include the standard pattern of the project output but the products made by the former students. Through examples, students can clarify the heterogeneous and complex issues that need to be explored. In addition, students are guided by the teacher to define the type of problem, structure, and direction of the solution. To activate ideas and creativity, students will need to negotiate within a group and solve the problem by questioning, imagination, and expansion.

The second stage is for product creation (plan, prototype, and product construction). In this stage, creativity and innovative thinking are iteratively blended. The product-oriented plan specifies how each group clarifies the situational problem, conducts further activities to advance the improvement, and develops the idea into a creative conceptual prototype. Based on the prototype, improvements and optimization links are constructed, and scheme adjustments and product tests are conducted with discussion. The process of optimization involves iterative adjustments of product solutions, analyses of the utility of prototypes arising from the inquiry activities, and timely discovery of their feasibility and effectiveness. After thorough testing and optimization, the final product is formed.

The third stage is display promotion (presentation and price). Through the integration of resources from various perspectives, students understand the product value of the market, society, and environment, so that they can present the product of the group’s project in a unique way for business promotion. Then, identify the rules of the market and try to price the product. The learning of AI technology and principles ultimately serves application and practice. Therefore, when students are consciously taught to transform their creativity into innovative thinking, they will be able to accumulate experience, analyze goals, execute works, and eventually approach completion. As a result of project creation and design, students gain more knowledge about business and markets, as well as consider the value and social significance of their ideas, which is conducive to innovation and entrepreneurship education.

### Research question and hypotheses

This paper tried to examine the effect of PBP in AI education. Specifically, we compared PBP with teacher-direct instruction and seek to find out whether PBP is more effective in promoting students’ project management skills, learning performance creativity, and innovative thinking. According to the research questions, four hypotheses were proposed as follows.

The first hypothesis was that PBP would promote students’ project management skills in AI courses. This result was expected because project management skills emphasize teamwork and project output (Birnberg, 1998; van Rooij, 2009), and PBP provides students with a product-oriented guide to the complete project planning and implementation process, which might develop basic project management skills with clear goals for the project.

The second hypothesis was that PBP would promote students’ learning performance. This result was expected because PBP provides a specific target for students to create meaningful products, which might help students to understand AI knowledge and procedure more deeply so that they would probably learn better (Despoina and Aikaterini, 2015).

The third hypothesis is that PBP would enhance students’ creativity. This result was expected because creativity emphasizes an intellectual quality that can produce novel, unique, socially meaningful, or personally valuable products (Lin, 2018); thus, students’ creativity could be triggered in PBP.

The fourth hypothesis is that PBP can improve students’ innovative thinking. This result was expected because innovative thinking is mostly characterized by the promotion of thinking outcomes, with emphasis on the feasibility of ideas or products (Fagerberg et al., 2005; Jones, 2015). Therefore, students’ innovative thinking is likely to be enhanced by value assessment and opportunity identification in PBP.

## Materials and methods

### Participants

The experiment was conducted in an information technology course of a high school in southeast China and lasted for 3 weeks. A total of 209 students in the tenth grade participated in the study. Among them, 107 students (i.e., 59 boys and 48 girls) were assigned to the treatment group with PBP pedagogy, and the other102 students (i.e., 56 boys and 46 girls) were assigned to the control group with direct instruction. The participants took two sessions of AI courses each week, and they can go to the lab anytime by appointment to complete their products. Both groups of students had no previous experience in AI courses. According to the results of the pretest on students’ creativity and innovative thinking, students in both groups have similar levels.

### Research design

In order to investigate the effectiveness of PBP in AI courses, this study compared the effects of PBP with those of direct instruction on four dependent variables (i.e., project management skills, learning achievement, creativity, and innovative thinking). Among these four dependent variables, the project management skills of the students were measured by a project evaluation scale containing five dimensions (i.e., topic, plan, tool, process, and product) from three-party perspectives (i.e., self-evaluation, peer evaluation, and teacher evaluation), which demonstrate how students improve project management through collaboration and inquiry. The learning achievement of the students was evaluated by the standard test offered in the textbook, which can be used to test students’ AI knowledge. The creativity of the students was evaluated by Williams’ Creativity Assessment Packet (Williams, 1980) based on four dimensions (i.e., curiosity, risk-taking, challenge, and imagination). The innovative thinking of students was evaluated by the General Innovation Skills Aptitude Test (General Innovation Skills Aptitude Test, 2000) with four dimensions (i.e., decision-making, feasibility, practicality, and validity). All of these instruments have been used in previous research and had good reliability.

### Procedure

In the experiment, the content of the course was about *Data and Computing* of *Artificial Intelligence and Its Application*. The teacher in charge of this course was a qualified teacher with 7 years of teaching experience and strong motivation. Participants in both the treatment group and the control group were taught by the same teacher and teaching assistant, and they were learning the same contents from identical textbooks. The only difference between these two groups was the pedagogy adopted.

**FIGURE 3:**
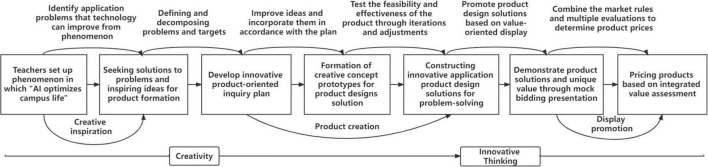
Specific implementation path of the product-based pedagogy AI curriculum.

In the treatment group, the product of the PBP AI course is presented as an innovative application of product design solutions. With the phenomenon “AI technology empowers campus life,” the teacher guides students to identify problems diversely such as inefficient manual temperature measurement at the school gate or crowded checkout at the canteen. Through group discussions and creative stimulation, teachers help students define and break down problems, explore AI technologies on the platforms, and enable creativity to grow. The students can experiment with multiple solutions to the same problem and negotiate the construction. For instance, a temperature measurement problem can be solved by using face recognition as the core and incorporating different technologies such as infrared temperature measurement or infrared thermal imaging. Through decision aggregation, the group stimulates ideas and creativity in product formation, which then leads to the determination of the final inquiry theme, and the development of the inquiry plan oriented toward the innovative application of the product. Students use examples of product solutions to improve their integrated ideas, ultimately developing a conceptual prototype of an innovative product design solution. Research, practical investigation, and feasibility analysis lead to iteration and revision of the product design, so students can test its feasibility and effectiveness, and finally create a prototype. Based on the phenomenon, teachers organize activities such as “campus bid simulations,” in which students present their product proposals from the perspectives of market, social and environmental values, reflecting the unique value of the product. Based on the feedback from other groups and the teacher, students priced their products considering the market rules and submitted a description of the product’s price. Using a combination of the evaluation dimension, the teacher ranked the final product. Figure 3 illustrates the specific implementation path of the PBP AI curriculum.

On the contrary, in the control group with direct instruction, students mainly learn through imitation, without emphasis on the creation of a product. Using the AI platform, the teacher explains how to use different AI technologies and conducts step-by-step demonstrations to enable students to master the corresponding theoretical and technical operations through imitation. For example, when teaching face recognition technology, the control group learned face recognition principles mainly by replicating the process: the teacher first explained the main component of the composition, and then students are guided to import photos for machine recognition and repeat the steps that are demonstrated by the teacher to experience and understand the face recognition technology.

### Measure instrument

#### Project management skills

Project evaluation scales include self-evaluation and peer evaluation, whereas teachers score students’ work based on the whole teaching session and the evaluation scale and finally use the average value of the three groups’ evaluations as an evaluation of the learning group’s output. The project evaluation scale contains five dimensions, such as topic selection (whether the topic has application value and innovative value), planning and design (whether it can accurately analyze the needs of the project), tools and methods (whether to carry out independent learning and collaborative learning around the project), steps and process (analyze whether the innovative application of artificial intelligence and its typical cases are complete), and the product and report (whether there is a correct understanding of the social impact of artificial intelligence). Using the reliability analysis, Cronbach’s alpha was 0.812. Also, the KMO value of the questionnaire was 0.839.

#### Learning achievement

The test of AI knowledge adopts the chapter test questions based on the textbook, covering the development and application of information technology, intelligent processing, AI applications, pattern recognition, the concept and characteristics of AI language, the impact of AI on human beings, the concept of AI, and other knowledge related to AI, through seven choice questions and three judgment questions to test students’ mastery of AI knowledge in the project learning process. Questions cover the theory and development of artificial intelligence and the future, with one point for each. According to the reliability analysis, Cronbach’s alpha was 0.797. The questionnaire had a KMO value of 0.784.

#### Creativity

Regarding the questionnaire on the creativity of students, this research is based on Williams’ Creativity Assessment Packet (Williams, 1980). The measure yielded a total creativity score and four sub-scores: curiosity, imagination, challenge, and risk-taking, which were derived from student self-responses to the statements scored on a Likert-type scale. The CAP was chosen because the four dimensions of the scale are important thinking characteristics and personality traits in the development of human creativity, and it is often used as a predictor of a person’s creative potential and level. CAP has some drawbacks; however, it cannot be recommended as an adequate assessment of the complex dimensions of creativity because its content validity is undermined by poor item and scoring definition. This study combined the four dimensions of the scale to revamp the pretest questionnaire for simplicity, ease, and validity, and 16 questions were revised. The response options range from 1 (non-conformance) to 3 (completely conforming). During and after the experiment, the same survey was administered to all students; 209 questionnaires were collected in the experiment. According to the reliability analysis results, Cronbach’s alpha for the new version of the questionnaire is 0.81. The KMO value is 0.785.

#### Innovative thinking

Based on the literature, it was found that the assessment of innovative thinking lacks a universally recognized evaluative tool. This study refers to the General Innovation Skills Aptitude Test (General Innovation Skills Aptitude Test, 2000), which combines four dimensions of decision-making, feasibility, practicality, and effectiveness. It improves awareness and understanding of the skills, attitudes, and behaviors that individuals and organizations need to innovate, as well as assessing the innovation skills needed by individuals and organizations to help them match innovation skills with their needs, and is therefore relevant in this study to test students’ innovation skills in product design. However, GISAT is only used in some countries to test innovation skills and is not universally accepted by academia. In addition, the study refines the four directions of thinking definition and classification in the thinking linkage model (Zhan et al., 2019), including relationship building, innovative thinking, follow-up and implementation, and risk control, and revised the questionnaire on innovative thinking assessment by integrating the innovation tendency scale with a total of 16 questions, with response options ranging from 1 (not conforming) to 3 (completely conforming). Based on the reliability analysis results, Cronbach’s alpha was 0.859. The KMO value for the questionnaire was 0.756.

### Data analysis

In this study, we took two steps to conduct data analysis by using SPSS. First, the normality statistics were calculated for the four dependent variables (i.e., project management skills, learning achievement, creativity, and innovative thinking). Then, two sets of independent-sample *t*-tests were used to test the learning effects of PBP by comparing the differences between the treatment group and control group on project management skills and learning achievement. Another two sets of ANCOVAs were employed to examine students’ creativity and innovative thinking by using the pretest score as a covariate.

## Results

### Project management skills

Independent-sample *t*-tests were conducted on the experimental data of each dimension of project management skills. A significant difference existed between the treatment and control groups of product scores shown in Table 2, *t* = 9.581, *p* < 0.001, and the effect size (based on Cohen’s d) was 0.549. In the integrated multi-subject evaluation, the treatment group’s scores are notably higher than those of the control group. The treatment group conducts product-oriented inquiry around artificial intelligence technology during learning activities to better meet product evaluation criteria.

**TABLE 2:** Means and standard deviation of students’ project management skills in treatment group and control group.

Dimension	Treatment group (*N* = 18)	Control group (*N* = 18)	*t*	*p*	*df*	Effect size
	*M* (*SD*)	*M* (*SD*)				
Self-evaluation	92.06 (2.127)	87.61 (1.819)	6.736	<0.001	34	0.419
Peer evaluation	88.33 (2.086)	83.28 (1.638)	8.086	<0.001	34	0.485
Teacher evaluation	89.06 (2.287)	83.72 (2.109)	7.273	<0.001	34	0.446
Product score	89.81 (2.678)	84.87 (2.685)	9.581	<0.001	106	0.549

### Learning achievement

In the independent-sample *t*-tests on AI knowledge in Table 3, there were no significant differences between the treatment group and control group, indicating that direct instruction and PBP are probably equally effective in students’ AI knowledge acquisition.

**TABLE 3:** Means and standard deviation of students’ learning achievement.

Dimension		AI knowledge	*t*	*p*	*df*	Effect size
Control group (*N* = 102)	*M* (*SD*)	7.95 (0.705)	−1.082	0.281	205	0.075
Treatment group (*N* = 107)	*M* (*SD*)	7.84 (0.761)				

### Creativity

Means (and SDs) for the students’ creativity before and after the treatment are shown in Table 4. In order to examine the differences between the experimental and control groups in students’ creativity, analysis of covariance (ANCOVA) was used, with pretest scores as a covariate. First, we checked whether the ANCOVA assumptions were met in the analysis of covariance using the Statistical Package for Social Sciences (SPSS version 26.0). The adjusted means (and SEs) for the two groups of creativity’s dimensions and total score are shown in Table 4 as well. It can be seen that in the experimental group, dimensions like curiosity (*F* = 6.260, *p* < 0.05, eta^2^ = 0.029), risk-taking (*F* = 13.358, *p* < 0.001, eta^2^ = 0.061), imagination (*F* = 26.887, *p* < 0.001, eta^2^ = 0.115), and the total score (*F* = 14.555, *p* < 0.001, eta^2^ = 0.066) are significantly higher than the control group. Results indicate that most creativity dimensions improved significantly under PBP except the challenge dimension.

**TABLE 4:** Descriptive statistics of students’ pretest and posttest scores and ANCOVA summary of creativity.

Factor	Group	Before treatment		After treatment		Univariate ANCOVA			
		Mean	*SD*	Mean	*SD*	Mean (adjusted)	SE	*F*-value	eta^2^
Curiosity	Treatment	9.02	1.427	9.56	1.183	9.56	0.144	6.260*	0.029
	Control	9.57	1.193	9.05	1.748	9.04	0.147		
Risk-taking	Treatment	8.75	1.190	9.33	1.097	9.33	0.117	13.358*	0.061
	Control	8.61	1.329	8.73	1.329	8.71	0.120		
Challenge	Treatment	9.36	1.456	9.31	1.450	9.31	0.129	2.818	0.011
	Control	9.38	1.347	8.99	1.183	8.98	0.132		
Imagination	Treatment	8.86	1.751	10.17	1.444	10.16	0.170	26.887*	0.115
	Control	10.18	1.440	8.90	2.027	8.90	0.175		
Total score	Treatment	36.51	3.717	37.96	3.412	37.96	0.400	14.555*	0.066
	Control	38.12	3.341	35.77	4.788	35.77	0.410		

There were 107 students in the treatment group and 102 students in the control group. ANCOVA, analysis of covariance.

*p < 0.05, significant p-value for ANCOVA and Bonferroni’s multiple comparisons test; eta^2^, effect size of ANCOVA (partial eta squared).

### Innovative thinking

Similarly, we use analysis of covariance (ANCOVA) to examine the innovative thinking between the experimental and control groups. SPSS was used to ensure that ANCOVA assumptions were met in the analysis of covariance. The means (and SDs) and the adjusted means for the two groups of students’ innovative thinking are shown in Table 5. It is evident that the treatment group performed significantly better than the control group on the dimensions of decision-making (*F* = 30.198, *p* < 0.001, eta^2^ = 0.128), feasibility (*F* = 55.249, *p* < 0.001, eta^2^ = 0.211), practicality (*F* = 46.557, *p* < 0.001, eta^2^ = 0.184), effectiveness (*F* = 40.092, *p* < 0.001, eta^2^ = 0.163), and the total score (*F* = 56.644, *p* < 0.001, eta^2^ = 0.216). The results indicate a significant improvement in innovative thinking.

**TABLE 5:** Descriptive statistics of students’ pretest and posttest scores and ANCOVA summary of innovative thinking.

Factor	Group	Before treatment		After treatment		Univariate ANCOVA			
		Mean	*SD*	Mean	*SD*	Mean (adjusted)	SE	*F*-value	eta^2^
Decision-making (relationship establishment)	Treatment	9.93	1.494	10.63	1.508	10.62	0.171	30.198*	0.128
	Control	9.81	1.612	9.27	2.001	9.27	0.175		
Feasibility (innovative ideas)	Treatment	8.87	1.756	10.14	1.610	10.13	0.165	55.249*	0.211
	Control	8.55	1.978	8.37	1.802	8.37	0.169		
Practicality (implementation)	Treatment	8.07	1.703	10.14	1.557	10.14	0.180	46.557*	0.184
	Control	8.28	2.022	8.39	2.126	8.38	0.184		
Effectiveness (risk control)	Treatment	9.33	1.682	10.36	1.538	10.365	0.167	40.092*	0.163
	Control	9.10	1.760	8.85	1.890	8.852	0.171		
Total score	Treatment	36.07	5.280	41.32	5.312	41.31	0.593	56.644*	0.216
	Control	35.75	5.767	34.92	6.863	34.92	0.607		

There were 107 students in the treatment group and 102 students in the control group. ANCOVA, analysis of covariance.

*p < 0.05, significant p-value for ANCOVA and Bonferroni’s multiple comparisons test; eta^2^, effect size of ANCOVA (partial eta squared).

## Discussion

### Product-based pedagogy helps to promote students’ project management skills

The first hypothesis that PBP would promote students’ project management skills was supported. As PBP is a special type of PBL, especially within the 7P model, it incorporates steps that promote active learning, engagement, interaction, and the ability to produce the desired product (Birnberg, 1998; van Rooij, 2009; Ganefri, 2013). Students in the PBP group have many opportunities to coordinate the project process, identify real problems that need to be solved, and transform the product as the goal of decomposition, in order to create a prototype of ideas and achieve final product formation through iteration and adjustment, customized to meet the values of the product. By focusing on clear project goals and specific product outcomes, students achieve a common goal of product design in a collaborative learning environment, thus effectively developing project management skills and promoting leadership (Loo, 1996; Burke and Barron, 2014). When it comes to product shaping, the 7P model emphasizes the estimation of product value and pricing as a method to assist students in AI courses in planning and designing products from the perspective of managing budgets, resources, and performance (Shariff et al., 2011).

### No significant difference was found in students’ learning performance between product-based pedagogy and direct instruction

Contradicting our expectations, the second hypothesis was not supported, and results indicated no significant difference was found in students’ learning performance between PBP and direct instruction. As claimed by previous studies, direct instruction could be very efficient in knowledge delivery (Hattie, 2008; Stockard, 2010; Flynn et al., 2012), because the one-way knowledge delivery process made by lectures and imitations is logical and systematical, which benefits students’ knowledge mastery (Heward and Twyman, 2021). Comparatively, PBP may be time-consuming for knowledge delivery, because students needed to learn by inquiry during the project; thus, the knowledge absorption could be fragmented. However, PBP has the advantage of enhancing the depth of the learning process and developing students’ emotional, cognitive, and psychomotor abilities (Despoina and Aikaterini, 2015). The tangible product also made the knowledge learned more embodied, comprehensible, and applicable. In PBP, knowledge is absorbed subliminally into all aspects of product design. The high-school AI course emphasizes a hierarchical decomposition of the working processes of complex intelligence with the aid of real-life examples (Lu et al., 2021). Because direct instruction can promote students’ mastery and absorption of AI knowledge efficiently, PBP can allow students to integrate their knowledge into the process of product design and enhance deeper learning. Both pedagogies have their benefit, which might be the reason for no obvious difference between them in terms of learning performance.

### Product-based pedagogy helps to improve students’ creativity

The third hypothesis that PBP promotes valid improvement in creativity was partially supported. Cross-sectional analysis of scores for each dimension (i.e., curiosity, risk-taking, challenge, and imagination) and the total scores of creativity revealed that students’ total level of creativity in the treatment group is significantly superior to those in the control group. Especially, the dimensions of curiosity, risk-taking, and imagination have been significantly improved, whereas no significant difference was found in the challenge dimension.

Risk-taking stresses the ability to confront mistakes or criticism and maintain one’s opinions while being able to anticipate versatility of thinking (Jia, 2008). Through the product iteration scaffold, students under PBP can make adjustments to the program by communicating content that is doubtful but form their understanding and then dare to try with trial and error. The perspective of curiosity consists of seeking out new things and situations, gaining insights from observations of particular phenomena, and investigating various explanations for why certain things happen (Zhang et al., 2012). As shown by treatment groups that examined artificial intelligence technology, this well describes the process of solving problems using curiosity. Conversely, the lack of curious exploration by the control group tends to cause a disconnection between learning outcomes and real-life problem-solving. Students in the PBP programs have the highest scores on imagination, and the process of creation is inherently the birth of new ideas, the ability of individuals to generate results from any kind of thinking (Zhou, 2015). In PBP, product-oriented learning enables students to pose questions and solve problems in imaginative ways, generate ideas from existing experiences, and develop their ideas (Pritzker and Runco, 2011). The lack of product guidance in the control group prevented the participants from conceptualizing how specific thinking would result, so they were more limited to simple tasks, and their imagination was insufficient to unleash creativity.

Despite the third hypothesis, which predicted that PBP would boost creativity in every dimension, we found no significant differences between the pretest and posttest of the challenging dimension in the treatment group. According to Young (2003), challenging learning is a process of constant challenge and transcendence in which students move through a cycle of challenge, action, feedback, and reflection. In parallel, the process is an upward spiral, where an additional challenge is introduced after completing a cycle, and students become capable of moving beyond their current skill levels; therefore, challenge development cannot be achieved overnight but requires a longer period.

### Product-based pedagogy helps to improve students’ innovative thinking

The fourth hypothesis, that PBP can promote all dimensions of innovative thinking, was well supported. Innovation involves transforming an idea into a useful product or system (Pavitt, 2003), that is, the process of putting creative ideas into practice (Rickards, 1985; Fjortoft et al., 2018). Students under PBP are product-oriented, and after generating product prototypes through creative stimulation, they can realize new, unique, and meaningful ideas, consider their feasibility and value through product iterative design, and conduct value assessment and opportunity identification, in which innovative thinking is well trained. Cross-sectional analyses of the two groups and vertical analyses of the pre- and pro-test scores of the treatment group showed that PBP significantly boosted all dimensions of innovative thinking.

Among the four dimensions, decision-making has the most prominence, focusing on the organization of knowledge in the group from a relationship-building perspective, along with an emphasis on the importance of individual effort in the formation of group decisions (Amabile and Pratt, 2016). The main component of innovative thinking training is decision-making, and the students under PBP divide labor within the group and examine all possible scenarios before deciding. Therefore, students had training in decision-making by discussing and choosing the best way to resolve the key issues that arose and contributing a convincing solution to promote the product design (Shi et al., 2018). There was also an improvement in the feasibility dimension, with students able to analyze AI technology and information processing from the standpoint of practicality and market acceptance of the technology and promote the product in terms of solving practical problems and offering commercial value. The feasibility component of technological innovation emphasizes the relevance of products and ideas, and it seeks results that are recognized and beneficial (Pan, 2019), which is encouraged as part of the PBP. In contrast, control group students had a weaker understanding of feasibility because they did not have product-oriented market value guidance, and the final learning outcomes were not always expressed in the form of a product. Students in the treatment group were proficient at weighing the originality, practicality, and social value of their outcomes through the presentation of the 7P model, which emphasized social satisfaction and recognition that contributed to the commercial value of the product. Innovation and creativity are characterized by the balance between novelty and efficacy (Runco, 2014), which is a remarkable reflection of the transformation of the two types of thinking under PBP.

In terms of practicality, the focus is on the value added as the goal, emphasizing market demand and application orientation for practical value (Fila et al., 2012). The primary focus of this project is to explore AI applications and information processing, given that students’ understanding of the business value and marketing skills are still superficial. Hence, practical training should expose students to projects with real market value and social significance, and more attention should be given to the product design and creation process for potential market and opportunity identification.

## Implication

AI has significant disruptive potential in the form of speed, breadth, and depth. New technology is the product of innovation, and it is also one of the greatest forces for creativity and innovative thinking education. Society attaches more importance to cultivating innovative talents as the driving force for economic growth in the twenty-first century. Students will gradually learn more about business and market knowledge as they age so that they can consider the market value and social significance of their product creation and put their ideas into practice. As part of this process, it enables students to gradually adapt to the needs of society and cultivate innovative talents for industry, academia, and research to meet the needs of future social-economic development and technological reforms. The high-school students’ creativity and innovative thinking should be nurtured at this stage, and we should drive students to develop better from internal and external motivators. In terms of knowledge learning and thinking training, the integration of PBP into high-school AI education has practical and theoretical significance.

The 7P model provides a feasible example for integrating PBP into high-school AI education. Using the phenomena of AI development in life, the course guides students to discover the actual problems that need solving, decompose and transform the problems, and then clarify learning objectives during follow-up learning. Project plans are continuously adjusted based on the problem and product orientation, generating prototypes of AI-related products. In AI education, the aim should not just be mastery of knowledge and skills, but also to incorporate creativity, innovative thinking, and product-oriented thinking, which are important in pedagogy.

This article proposes a pedagogy for high-school AI courses that make product creation a priority to cultivate knowledge and train thinking. Based on the experimental school’s situation, we propose a high-school AI curriculum that is based on PBP, for students to participate easily in the process of product creation, and learn effectively to develop creativity and innovative thinking. Having said that, not all projects end up as products, so this pedagogy may not be appropriate for projects that address political or ethical issues or where student activities tend to be learning-oriented rather than construct-oriented. Though there is some controversy over the application scope and the role of products in the learning process, we believe that the emphasis on product orientation in PBP can help cultivate students’ creativity and innovative thinking, which could also serve as a model for the pedagogy of product-oriented entrepreneurship and maker education.

## Limitations and future study

The purpose of this research is to demonstrate the effect of PBP on high-school AI courses and also to provide materials for future study. Certain difficulties arose in the design of this study, and there were some limitations. First, because the experiment was conducted by the same teacher using different pedagogies for the treatment group and the control group, it might cause individual preferences on the instructional guidance and affect the experimental outcome. In addition, change during thinking training was not measured. Creativity and innovative thinking can be improved through specific teaching methods and activity design, but it is hard to identify the point at which the links begin to make an effect. Therefore, in response to these limitations, some potential future directions were suggested for follow-up research.

First, it might be interesting to get more insights into the collaboration process of PBP. Attention must be paid to the individual creative concept formed by the divergence of thinking, and the learning task can be set up in layers so that students can gradually transform from individual activities to collaborative learning. Full play must be given to the effectiveness of prior knowledge and creativity in individual behaviors, and promote subsequent decision-making aggregation. Alternatively, it could be referred to as participatory creativity, which introduces opportunity, equity, and a dynamic reconfiguration of innovation and invention from individual or group presentations (Clapp, 2016; Clapp and Jimenez, 2017).

Second, it is important to investigate the mechanism of supporting the transformation from creativity to innovation and further develop the pedagogy for facilitating learning. In the process of cultivating innovative talents, we need to guide students to design creative products and fit the product into the actual market. It is essential for a product with a creative imagination and convergent innovation that could accelerate the market, and also through market feedback to amend creativity. Therefore, the realistic pursuit of student work should be emphasized in the innovation process.

## Data availability statement

The original contributions presented in this study are included in the article/supplementary material, further inquiries can be directed to the corresponding author/s.

## Ethics statement

The studies involving human participants were reviewed and approved by the South China Normal University. Written informed consent to participate in this study was provided by the participants’ legal guardian/next of kin.

## Author contributions

ZZ identified research ideas, designed and facilitated this research, wrote the draft, and made substantial revisions to this work. WS conducted the experiments, analyzed the data, wrote the draft, and revised the manuscript. WL assisted with data collection and provided advice on revisions. All authors contributed to the article and approved the submitted version.
